# Cost Analysis of BEST in CLASS-Elementary: Average Cost per Teacher-Student Dyad and Marginal Cost per Additional Student

**DOI:** 10.1007/s11121-026-01938-8

**Published:** 2026-06-05

**Authors:** K. S. Sutherland, M. A. Conroy, A. Blandin, K. L. Granger, Isabel Li

**Affiliations:** 1 Virginia Commonwealth University, Richmond, VA USA; 2 University of Florida, Gainesville, FL USA; 3 Vanderbilt University, Nashville, TN USA

**Keywords:** Cost analysis, School-based prevention

## Abstract

This study used the Ingredients Cost Method (Levin & McEwan, 2001) to calculate the average cost of implementing BEST in CLASS-Elementary, a Tier 2 program that trains and coaches teachers to provide support to focal students who have unwanted problem behavior. Data were used from kindergarten to third-grade classrooms in a multisite randomized controlled trial. We describe the average cost of implementing BEST in CLASS-Elementary per teacher-student dyad as well as the marginal cost of adding up to two additional students in classrooms receiving the training and coaching. Data from 136 teacher student dyads (*N* = 63 teachers) were used in the current study. The average cost to implement the program, including teacher recruitment costs, was $2,866 per teacher-student dyad in a classroom, and the marginal cost of adding an additional focal student per treated classroom was $101. The actual intervention accounted for less than half of the total costs and coaches accounted for the highest investment (almost half of the total costs). Limitations of the current study as well as implications for future research in this area are discussed.

Approximately 20% of students in the USA have social, emotional and behavioral (SEB) needs that place them at risk for developing emotional/behavioral disorders (EBD; Ringeisen et al., 2020), and public schools report that 19% of their students receive some type of school-based mental health service (NCES, 2024). However, schools continue to struggle to meet the complex SEB needs of these students, and these challenges have been exacerbated by a number of factors, including the COVID-19 pandemic (Naff et al., 2022), teacher shortages (Bettini et al., 2017; Yarrell, 2022) and, most recently, federal cuts to programs supporting student SEB needs in schools (Turner, 2025). Even before these federal cuts to programs were enacted, over half of public schools reported not having sufficient staff or funding to provide the SEB supports needed for these students (NCES, 2024). Although evidence-based programs (EBP) exist that schools can implement to support students’ SEB needs (e.g., *Incredible Years*, Webster-Stratton et al., 2004; *First Step to Success*, Walker et al., 1998; *Promoting Alternative Thinking Strategies (PATHS)*, Conduct Problems Prevention Group, 2010; Greenberg et al., 1995; *Good Behavior Game*, Kellam et al., 2008), understanding the costs to schools of implementing EBPs, particularly for students who need additional supports, is heightened given the clear need for such programs coupled with limited (and decreasing) school resources available to support such programming. Indeed, detailing the costs (both monetary and time associated with training and implementation supports) of EBPs is an important determinant of whether EBPs are adopted (Lindstrom et al., 2023; Strayer et al., 2022). As such, the purpose of this study is to examine the costs of implementing an EBP, BEST in CLASS-Elementary (McCullough et al., 2022; Sutherland et al., 2020), in elementary schools that support teachers’ delivery of a targeted intervention to address the needs of students who have more intensive social, emotional, and behavioral needs.

BEST in CLASS-Elementary (Sutherland et al., 2020) is a Tier 2 intervention that trains teachers to increase both their frequency and quality of implementation of a core set of evidence-based practices (e.g., supportive relationships, rules, precorrection, opportunities to respond, praise, home-school partnerships) with up to three focal students per classroom who have been identified as demonstrating elevated problem behaviors. Following training in the program, teachers are provided practice-based coaching (see Snyder, 2022) for approximately 15 weeks to support their implementation of the practices with fidelity. Adapted from the early childhood version of BEST in CLASS – Pre-K (Conroy et al., 2019; Sutherland et al., 2018), teachers who implement BEST in CLASS-Elementary have had reductions in burnout (McCullough et al., 2022), improvements in self-efficacy (Sutherland et al., 2026), and increased positive relationships with their focal students (Conroy et al., 2026; Sutherland et al., 2020). At the same time, focal students have shown reduced problem behaviors (Conroy et al., 2026; Sutherland et al., 2020). As such, those working in schools that might adopt BEST in CLASS–Elementary should be aware of the costs involved in implementation.

Understanding the costs of implementation of EBPs in schools is made challenging given the levels, or tiers, of programming available to schools. Schools tend to implement SEB programs within multi-tiered systems of support (MTSS) made common by Positive Behavioral Interventions and Supports (PBIS; Sugai & Horner, 2009). This framework helps guide intervention supports at the Tier 1 (universal) level; when students are non-responsive to Tier 1 supports, they receive more intensive Tier 2 supports, and students non-responsive at the Tier 2 level receive even more intensive, individualized supports at the Tier 3 level. Much of the cost analysis work that has been done on school-based SEB programming has been done with PBIS implementation as well as interventions at the Tier 1, or universal, level, with less research at the Tier 2 or Tier 3 levels.

In terms of Tier 1 interventions, Kuyken et al. (2022) examined costs associated with implementing a school-based mindfulness intervention (MYRIAD) in 42 schools in the UK finding the cost per student was £71 ($95 in 2025 US dollars). Several cost analysis studies of PATHS, a universal social-emotional learning program, have also been conducted. Berry et al. (2016) conducted a cost analysis of PATHS within a large RCT in the UK using a cost-consequence analysis. The average cost per school was £12,666 per school ($16,958 in 2025 in US dollars) and £139 per student ($186 in 2025 US dollars). In a similar large RCT in the United Kingdom Humphrey et al. (2018) found that the mean incremental cost of implementation of PATHS in comparison to business as usual (BAU) was £30 ($40 in 2025 US dollars).

There have been some cost analysis studies at the Tier 2 level, but many of these studies focused on analyzing costs in early care and education settings (Frey et al., 2019; Olchowski et al., 2007) rather than school settings. Olchowski and colleagues estimated costs for the child and teacher training of the Incredible Years program, finding that there was a per-child cost of $1,164 for the child intervention and an additional $289 for the teacher intervention, with a total cost of $1,454 per child. In another study Frey et al. (2019) found that the estimated average cost of First Steps implementation per child in an early care and education setting was $2,716, with an additional average cost of $719 for follow-up supports in kindergarten. The incremental cost of adding another child per classroom was $1,911 in preschool, with an additional cost for follow-up supports in kindergarten of $569.

These studies highlight that over the past 20 years, cost analysis studies have been conducted, which help potential adopters to make informed decisions about their investment. That said, schools continue to struggle to meet the SEB needs of their students and need to ensure their limited resources are used effectively, magnifying the need for schools to understand the cost of EBPs to support their students. It is also important for schools to best understand the marginal cost of adding students to an intervention once initial investments have been made (Frey et al., 2019), as these costs add up to real-world investments in the prevention of future SEBs. If schools are aware of the costs prior to adopting an EBP, they can ensure that they have the resources for adoption and implementation. In the current study, we used the Ingredients Cost Method (Levin & McEwan, 2001) to calculate the average cost of implementing BEST in CLASS-Elementary with teacher-student dyads in kindergarten through 3rd-grade classrooms. Specifically, the aims of the current study are to (1) describe the average cost of implementing BEST in CLASS-Elementary per teacher-student dyad, and (2) describe the marginal cost of adding an additional student within a classroom already receiving BEST in CLASS-Elementary training and coaching.

## Method

This cost analysis used data from a multisite cluster randomized controlled trial of BEST in CLASS-Elementary in two southeastern U.S. states. This research was conducted in four school districts and 20 elementary schools representing urban, rural, and suburban communities. Most (*n* = 19) of the schools were public, while one was a charter school. There was an average of 470 students per school, with 45% Black students, 32% White students, and 18% Hispanic students. Approximately 88% of the students in these schools were from disadvantaged socioeconomic backgrounds as reflected by free/reduced school lunch data. Classrooms within schools were randomly assigned to the BEST in CLASS-Elementary condition or a business-as-usual condition, and the BEST in CLASS-Elementary intervention condition was used as a data source for this study. For the current study, BEST in CLASS-Elementary was implemented during the 2018–2019, 2019–2020, 2021–2022, and 2022–2023 school years; year-end data collection in 2020 was impacted by the COVID-19 pandemic, and due to school closures, the program was not implemented during the 2020–2021 school year.

### Participants

The study team met with kindergarten to 3rd grade teachers in schools in the participating districts, where principals agreed that their teachers could participate. Teachers were eligible to participate in the study if (1) they taught kindergarten, first, second, or third grades, and (2) they had at least one student who met eligibility criteria for the study (see Conroy et al., 2026 for more detail about the student screening procedures as well as details about the student sample). Of 114 teachers who met these criteria, 63 were randomly assigned to the BEST in CLASS-Elementary condition. Of 237 eligible students, 136 were in BEST in CLASS-Elementary teachers’ classrooms. Data from these teacher-student dyads are used in the current study. Given the Tier 2 nature of the intervention, BEST in CLASS was designed to support up to three focal students per classroom.

### BEST in CLASS-Elementary Implementation

BEST in CLASS-Elementary trains and coaches teachers to increase their use of BEST in CLASS-Elementary practices with 1–3 focal students per classroom with high rates of problem behaviors, which place them at risk for EBDs. BEST in CLASS-Elementary teachers received 6-h of didactic training on implementing the practices, a manual that provides an overview of the practices (home-school partnerships, supportive relationships, rules, precorrection, opportunities to respond, praise) and the implementation process, and 14 weeks of practice-based coaching that emphasizes implementing the practices with focal students with frequency and high quality throughout the school day. The training provides teachers with detailed information about each BEST in CLASS-Elementary practice, modeling of the practices, video exemplars, and activities that encourage discussion among teachers and coaches about specific problem situations in their classrooms.

After teachers have completed the workshop, coaches and teachers meet weekly using a practice-based coaching framework (see Snyder, 2022). Each week, teachers and coaches collaborate to develop a weekly shared goal for the BEST in CLASS-Elementary practice (e.g., supportive relationships), and coaches support teachers with implementation. Coaches conduct structured observations and videorecording to collect data on the teachers’ use of the practices with the focal student during a teacher-led instructional activity, and use these data to provide supportive and constructive feedback to teachers on their use of the practice with focal students (for more detail on the BEST in CLASS-Elementary practice-based coaching, see Conroy et al., 2026; Sutherland et al., 2019).

### Cost Framework

The cost analysis was conducted from the perspective of the intervening organization, in this case a school. The total cost represents the combined cost to the school of all the inputs that were used to implement the intervention. Interventions often involve a broad range of inputs, including materials, recruitment incentives, labor, and facilities. The Ingredients Method (Levin & McEwan, 2001) involves multiplying the quantity and unit price of each input and then summing them together to arrive at a total cost. Specifically, the formula for an intervention’s total cost, C, is given by:

$$C =\sum_{i=1}^{I}{p}_{i}{q}_{i}$$

In this equation, ${q}_{i}$ is the quantity of input i and ${p}_{i}$ is the price of one unit of input i. The total cost of input i is the product of its unit price and the quantity of units used, ${p}_{i}{q}_{i}$. To arrive at the intervention’s total cost, we then sum the total costs of all inputs i=1,⋯,I. Intervention inputs that were obtained at zero price by the organization do not factor into an intervention’s cost. For example, an intervention may involve coaching that takes place in a classroom (a facility) that was provided at no cost. In this case, the cost analysis should note that that coaching facilities were freely available so that future implementers can accurately interpret the cost analysis. Sensitivity analyses can be used to show how intervention costs would change if, for example, coaching spaces entailed additional costs. More generally, sensitivity analyses can demonstrate how costs would vary across different settings, including variation in salaries, transportation costs, or facility costs.

Teacher time (e.g., time spent training) is an important input in many interventions. From a cost analysis perspective, the relevant price of a teacher’s time is the cost to the intervening organization of inducing the teacher to participate. This could include incentive payments to recruit teachers and/or payments to substitute teachers who filled in for teachers while they were training. In the present study, payment to recruit teachers included $400 in gift cards or checks, and this serves as a proxy for teacher participation cost and not necessarily the full opportunity cost of teacher time. Some previous cost analysis papers have alternately used teachers’ hourly compensation as the unit price of an hour of a teacher’s time (Barrett & VanDerHeyden, 2020; Frey et al., 2024). This would be the relevant cost if, for example, participation was mandatory and involved additional hours of work by the teacher. As with other inputs, cost analyses should carefully explain how teacher time is priced and conduct sensitivity analyses to variations in this price. In our cost analysis, we consider two cost calculations that treat the cost of teacher recruitment differently. Cost 1 excludes teacher recruitment costs altogether. This would be the relevant calculation for a setting in which teachers were already required to conduct professional development training and BEST in CLASS-Elementary was substituted for a previous program (assuming that the previous personal development training entailed the same teacher time commitment as BEST in CLASS-Elementary). Cost 2 includes teacher recruitment costs. This would be the relevant calculation for a setting in which BEST in CLASS-Elementary was offered to teachers as a voluntary training opportunity.

Input costs exclusively associated with program evaluation or research are excluded from the analysis (Crowley et al., 2018). These inputs are not necessary to conduct the intervention and therefore are not relevant for future implementation decisions.

Our results translate total costs into average costs per teacher-student dyad and the marginal cost of including an additional student per classroom; importantly, BEST in CLASS-Elementary was designed to support up to but not more than three focal students per classroom. The average cost per student is the total intervention cost divided by the number of treated students per classroom. The marginal cost of including an additional student is the incremental cost to include one more student in the intervention within an already-treated classroom. Marginal costs can differ from average costs if implementation costs do not scale linearly with the number of students in a classroom. For example, in BEST in CLASS-Elementary, the cost to recruit a teacher is an important component of the average cost. However, this cost does not factor into the marginal cost of including an additional student within an already-treated classroom, because that student’s teacher has already been recruited.

### Cost Measures

All costs were averaged across both state sites.

#### Personnel Wage and Fringe Benefit Rates

For the purposes of this study, a program manager external to the school is included to help support implementation of the BEST in CLASS-Elementary intervention in the school. This individual helped to coordinate research activities and implementation. For the BEST in CLASS-Elementary program manager, an annual salary was divided by the number of hours in a work year (2,080), to determine an hourly wage ($50 in state 1, $34 in state 2); fringe benefit rates were from project budget records. Hours spent by the BEST in CLASS-Elementary managers on recruitment, screening, and training were estimated retrospectively from project records and calendars showing these activities. Hourly wage and fringe benefit rates for coaches and fidelity coders came from payroll records.

#### Personnel Hours

Coaching was the most resource-intensive personnel position. Hours spent by coaches came directly from hourly time sheets, submitted prospectively on a bi-weekly basis throughout their involvement in the study. We used notes from supervision meetings, where interventionists were assigned to students, to identify intervention start and stop dates for each coach and then allocated hours across major activities (i.e., preintervention training, intervention, and cross-coach collaboration). Any hours incurred prior to the intervention start date were allocated to pre-intervention. Hours from the coach’s first to last intervention dates were assigned to intervention and cross-coach collaboration activities. One hour per week was allocated to cross-coach collaboration meetings.

#### Supplies

This category included supplies needed for pre-intervention and the intervention itself. Pre-intervention materials were student screening kits ($11 per teacher).

One kit is provided per teacher and is sufficient to screen up to five students in a classroom.

Intervention materials included BEST in CLASS-Elementary teacher manual and workbook ($30 per teacher in Site 1, $14 per teacher in Site 2), and coaching/observation manuals and forms ($60 per teacher in Site 1, $30 per teacher in Site 2). Quantities and unit costs came from project expense records and university procard/credit card receipts.

#### Mileage Reimbursement

The intervention reimbursed coaches for miles driven between the university and the schools. The reimbursement rate was $0.45 per mile in state 1 and $0.61 per mile in state 2. Mileage amounts and unit costs came from project expense records.

#### Facilities

All program activities took place inside the school and were made available at zero cost. Therefore, facilities were excluded from the analysis (Crowley et al., 2018). However, consistent with the ingredients method, the use of space represents a real resource cost. Districts implementing BEST in CLASS in settings without available space or technology should incorporate the value of facilities and equipment when estimating local implementation costs.

### Sensitivity Analysis

The largest cost category of BEST in CLASS-Elementary is personnel costs. Because wages vary widely across locations in the USA, the overall BEST in CLASS-Elementary cost is also likely to vary by location. We gauge the extent of this variation by simulating alternative program costs after adjusting for state-level variation in wages. Specifically, we scale personnel costs according to differences in mean hourly wages in each state relative to the mean hourly wage in our intervention states of Site 1 and Site 2 (Bureau of Labor Statistics, 2025). We assume that materials and transportation costs remain fixed across states.

## Results

### The Cost to Implement BEST in CLASS-Elementary

Table 1 summarizes the cost to implement BEST in CLASS-Elementary. The total implementation cost with 2 program managers, 24 coaches, 8 fidelity coders, and 63 teachers was $180,528. This implies an average cost per teacher-student dyad of $2,402 excluding teacher recruitment costs (Average Cost 1) or $2,866 including teacher recruitment costs (Average Cost 2). Going forward we will primarily focus our discussion on Average Cost 2, which includes recruitment costs, as this best reflects our implementation. Table 1 decomposes the overall cost by implementation phase. The actual intervention itself accounts for just under half (47.3%) of total costs. The remainder is accounted for by pre-intervention (19.8%), post-intervention support (18.3%), transportation (11.4%), and materials (3.2%).

**Table 1:** Cost of BEST in CLASS: by implementation phase

	Total cost	Average Cost 1	(% of total)	Average Cost 2	(% of total)
**Pre-intervention subtotal**	**$35,665.00**	**$566.11**	**(23.6%)**	**$566.11**	**(19.8%)**
Program managers	$15,169.50	$240.79		$240.79	
Coaches	$18,828.52	$298.87		$298.87	
Fidelity coders	$1,666.98	$26.46		$26.46	
**Intervention subtotal**	**$85,459.73**	**$893.45**	**(37.2%)**	**$1,356.50**	**(47.3%)**
Program managers	$7,584.75	$120.39		$120.39	
Coaches	$38,200.89	$606.36		$606.36	
Fidelity coders	$10,501.96	$166.70		$166.70	
Teachers	$29,172.12			$463.05	
**Post-intervention support subtotal**	**$33,052.01**	**$524.64**	**(21.8%)**	**$524.64**	**(18.3%)**
Program managers	$7,584.75	$120.39		$120.39	
Coaches	$25,467.26	$404.24		$404.24	
**Materials subtotal**	**$5,714.82**	**$90.71**	**(3.8%)**	**$90.71**	**(3.2%)**
Student screening kits	$802.23	$12.73		$12.73	
Teacher packet	$1,613.95	$25.62		$25.62	
Coach packet	$3,298.64	$52.36		$52.36	
**Transportation subtotal**	**$20,636.14**	**$327.56**	**(13.6%)**	**$327.56**	**(11.4%)**
Mileage payments	$20,636.14	$327.56		$327.56	
**Total**	**$180,527.70**	**$2,402.47**	**(100.0%)**	**$2,865.52**	**(100.0%)**

Prices are in 2024 dollars (intervention took place in years 2018–2022). Prices in different years were adjusted using the Personal Consumption Expenditure (PCE) index. Average Cost 1 excludes the cost of teacher recruitment

Table 2 decomposes the overall cost by input category. Focusing on Average Cost 2, the costliest component of the intervention is coaches, who account for 45.7% of the overall cost. Of this coaching cost, 22.8% includes pre-intervention costs (i.e., training), 46.3% includes intervention costs (i.e., direct coaching) and 30.9% includes support (i.e., supervision). The remainder of the overall cost is accounted for by teacher recruitment (16.2%), program managers (16.8%), transportation (11.4%), fidelity coders (6.7%), and materials (3.2%).

**Table 2:** Cost of BEST in CLASS: by input category

	Total cost	Average Cost 1	(% of total)	Average Cost 2	(% of total)
**Program managers subtotal**	**$30,339.01**	**$481.57**	**(20.0%)**	**$481.57**	**(16.8%)**
Pre-intervention	$15,169.50	$240.79		$240.79	
Intervention	$7,584.75	$120.39		$120.39	
Support	$7,584.75	$120.39		$120.39	
**Coaches subtotal**	**$82,496.68**	**$1,309.47**	**(54.5%)**	**$1,309.47**	**(45.7%)**
Pre-intervention	$18,828.52	$298.87		$298.87	
Intervention	$38,200.89	$606.36		$606.36	
Support	$25,467.26	$404.24		$404.24	
**Fidelity coders subtotal**	**$12,168.94**	**$193.16**	**(8.0%)**	**$193.16**	**(6.7%)**
Pre-intervention	$1,666.98	$26.46		$26.46	
Intervention	$10,501.96	$166.70		$166.70	
**Teachers subtotal**	**$29,172.12**			**$463.05**	**(16.2%)**
Intervention	$29,172.12			$463.05	
**Materials subtotal**	**$5,714.82**	**$90.71**	**(3.8%)**	**$90.71**	**(3.2%)**
Student screening kits	$802.23	$12.73		$12.73	
Teacher packet	$1,613.95	$25.62		$25.62	
Coach packet	$3,298.64	$52.36		$52.36	
**Transportation subtotal**	**$20,636.14**	**$327.56**	**(13.6%)**	**$327.56**	**(11.4%)**
Mileage payments	$20,636.14	$327.56		$327.56	
**Total**	**$180,527.70**	**$2,402.47**	**(100.0%)**	**$2,865.52**	**(100.0%)**

Prices are in 2024 dollars (intervention took place in years 2018–2022). Prices in different years were adjusted using the Personal Consumption Expenditure (PCE) index. Average Cost 1 excludes the cost of teacher recruitment

Table 3 displays the marginal cost of treating additional teachers or students. The marginal cost of treating an additional teacher is below the average cost because some costs do not vary with the number of teachers or students. Focusing on Cost definition 2 (which includes teacher recruitment costs), the Marginal Cost 2 of treating an additional teacher is $2,059, which is 28% lower than the Average Cost 2 per teacher. The marginal cost of an additional teacher is lower than the average cost per teacher because we assume that (i) no additional program managers are needed, (ii) no additional coaches need to be trained, and (iii) no additional fidelity coders need to be trained. If the program were to expand to the extent that an additional program manager, coach, or fidelity coders do need to be hired and trained, this would increase the marginal cost in the direction of the average cost per teacher.

**Table 3:** Marginal cost of BEST in CLASS: by input category

	Marginal Cost 1 of treating additional teacher	(% of total)	Marginal Cost 2 of treating additional teacher	(% of total)	Marginal cost of treating additional student	(% of total)
**Program managers subtotal**	**$0.00**	**(0.0%)**	**$0.00**	**(0.0%)**	**$0.00**	**(0.0%)**
**Coaches subtotal**	**$1,010.61**	**(63.3%)**	**$1,010.61**	**(49.1%)**	**$101.06**	**(100.0%)**
Pre-intervention	$0.00		$0.00		$0.00	
Intervention	$606.36		$606.36		$101.06	
Support	$404.24		$404.24		$0.00	
**Fidelity coders subtotal**	**$166.70**	**(10.4%)**	**$166.70**	**(8.1%)**	**$0.00**	**(0.0%)**
Pre-intervention	$0.00		$0.00		$0.00	
Intervention	$166.70		$166.70		$0.00	
**Teachers subtotal**			**$463.05**	**(22.5%)**	**$0.00**	**(0.0%)**
Intervention			$463.05		$0.00	
**Materials subtotal**	**$90.71**	**(5.7%)**	**$90.71**	**(4.4%)**	**$0.00**	**(0.0%)**
Student screening kits	$12.73		$12.73		$0.00	
Teacher packet	$25.62		$25.62		$0.00	
Coach packet	$52.36		$52.36		$0.00	
**Transportation subtotal**	**$327.56**	**(20.5%)**	**$327.56**	**(15.9%)**	**$0.00**	**(0.0%)**
Mileage payments	$327.56		$327.56		$0.00	
**Total**	**$1,595.57**	**(100.0%)**	**$2,058.62**	**(100.0%)**	**$101.06**	**(100.0%)**

Prices are in 2024 dollars (intervention took place in years 2018–2022). Prices in different years were adjusted using the Personal Consumption Expenditure (PCE) index. Marginal Cost 1 excludes the cost of teacher recruitment

The final column of Table 3 also displays the marginal cost of treating an additional student in a classroom of an already-treated teacher. To arrive at this marginal cost, we first identified which resources would vary if one additional student received BEST in CLASS-Elementary, versus which would remain invariant or fixed. We assumed the additional student would be at a school already participating in the intervention and that a trained coach and teacher would be assigned; thus, no additional school recruitment or coach training costs would be incurred. Further, screening kits come in one packet per classroom so no additional costs would be incurred by adding a student. Finally, no additional intervention materials or supplies would be needed by adding one student; thus, we consider these factors fixed. Coach support occurred in one meeting per week with the classroom teacher, as such approximately 15 min would be added to each coaching meeting per additional student. Incremental coach and teacher resources (e.g., hours per student) were the averages per student and were multiplied by the relevant wage and fringe rate for that personnel category to determine incremental personnel costs. The marginal cost is $101, which is the cost of coaching support for an additional focal student per classroom where the teacher is already receiving coaching support for another focal student. Finally, Table 4 presents costs separately by site to help illustrate differences across the two implementation contexts.

**Table 4:** Costs by site

	Total cost: FL	Average Cost 2: FL	(% of total)	Total cost: VA	Average Cost 2: VA	(% of total)
**Program managers subtotal**	**$18,058.93**	**$564.34**	**(17.3%)**	**$12,280.07**	**$396.13**	**(16.2%)**
Pre-intervention	$9,029.47	$282.17		$6,140.04	$198.07	
Intervention	$4,514.73	$141.09		$3,070.02	$99.03	
Support	$4,514.73	$141.09		$3,070.02	$99.03	
**Coaches subtotal**	**$46,883.77**	**$1,465.12**	**(44.8%)**	**$35,612.91**	**$1,148.80**	**(46.9%)**
Pre-intervention	$9,376.75	$293.02		$9,451.77	$304.90	
Intervention	$22,504.21	$703.26		$15,696.69	$506.34	
Support	$15,002.81	$468.84		$10,464.46	$337.56	
**Fidelity coders subtotal**	**$6,167.82**	**$192.74**	**(5.9%)**	**$6,001.12**	**$193.58**	**(7.9%)**
Pre-intervention	$833.49	$26.05		$833.49	$26.89	
Intervention	$5,334.33	$166.70		$5,167.63	$166.70	
**Teachers subtotal**	**$14,817.59**	**$463.05**	**(14.2%)**	**$14,354.54**	**$463.05**	**(18.9%)**
Intervention	$14,817.59	$463.05		$14,354.54	$463.05	
**Materials subtotal**	**$3,741.44**	**$116.92**	**(3.6%)**	**$1,973.75**	**$63.67**	**(2.6%)**
Student screening kits	$407.48	$12.73		$394.75	$12.73	
Teacher packet	$1,111.32	$34.73		$502.41	$16.21	
Coach packet	$2,222.64	$69.46		$1,076.59	$34.73	
**Transportation subtotal**	**$15,002.81**	**$468.84**	**(14.3%)**	**$5,779.14**	**$186.42**	**(7.6%)**
Mileage payments	$15,002.81	$468.84		$5,779.14	$186.42	
**Total**	**$104,672.35**	**$3,271.01**	**(100%)**	**$76,001.53**	**$2,451.66**	**(100.0%)**

Prices are in 2024 dollars (intervention took place in years 2018–2022). Prices in different years were adjusted using the Personal Consumption Expenditure (PCE) index

### Sensitivity Analysis

**Fig. 1:**
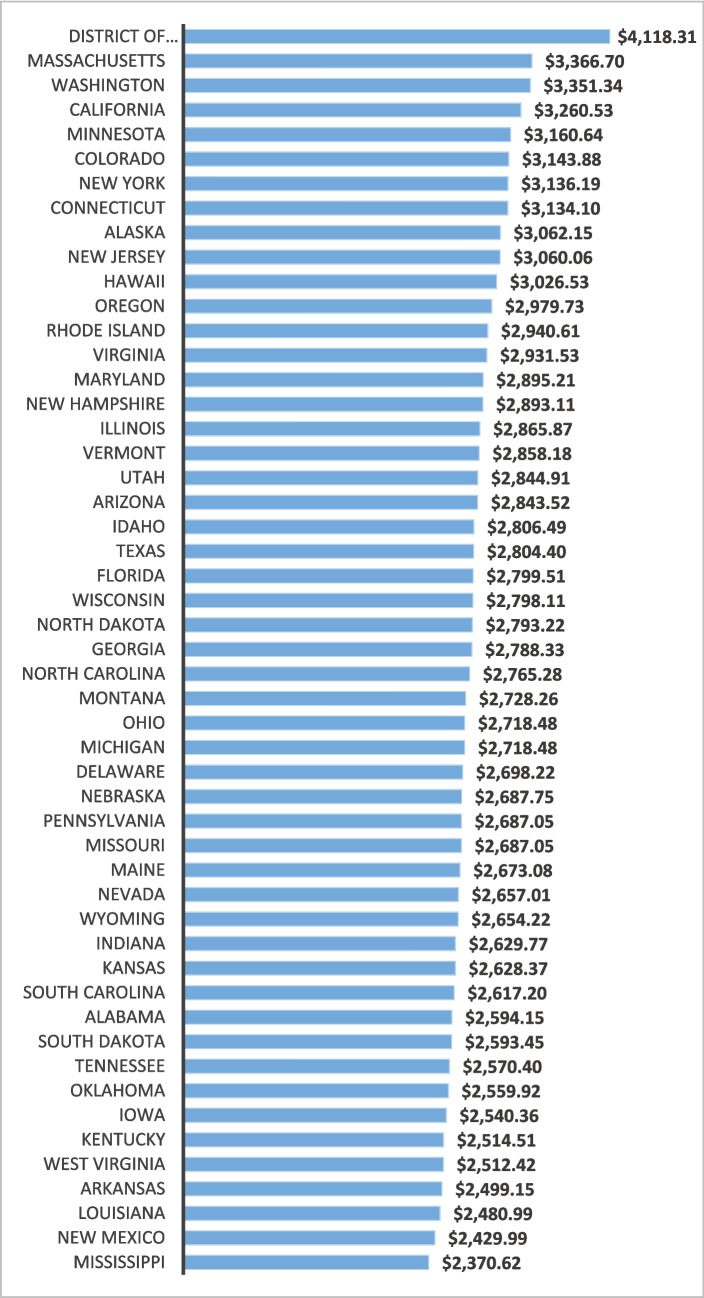
Estimated cost of BEST in CLASS-elementary by state

Figure 1 displays the simulated average cost per teacher-student dyad of BEST in CLASS-Elementary across the 50 U.S. states and the District of Columbia. To translate cost into different states, we multiplied our average cost by the mean wage of the state relative to the average of our two treatment states. The costs correspond to Average Cost 2 (including teacher recruitment costs). In the states with the highest mean wages (DC, Massachusetts, and Washington), the average cost per teacher is above $2,800 (compared to our average cost per teacher of $2,866). In the states with the lowest mean wages (New Mexico and Mississippi), the average cost per teacher is below $2,100. This analysis suggests that the average cost of implementation could vary by as much as $2,800 / $2,100 = 33% depending on local wages.

## Discussion

The purpose of the current study was to describe the average cost of implementing BEST in CLASS-Elementary per teacher-student dyad, as well as to describe the marginal cost of adding an additional student within a classroom already receiving BEST in CLASS-Elementary training and coaching. Using the Ingredients Method (Levin & McEwan, 2001) we determined that the average cost to implement the program, including teacher recruitment costs, was $2,866 per teacher-student dyad, with the actual intervention accounting for less than half of the total costs and coaches accounting for the costliest investment (almost half of the total costs). The marginal cost of adding an additional focal student per treated classroom was $101. Below we further discuss our findings, situating the costs within the existing literature; we finish with a discussion of the limitations of the current study as well as implications for future research in this area.

While it can be difficult to compare costs of implementing school-based EBPs due to the varying levels of intensity (i.e., tiers) as well as settings (i.e., early care and education settings; elementary schools) of the interventions, the cost to implement BEST in CLASS-Elementary appears to be in line with other programs. To illustrate, Olchowski et al. (2007) found per child costs of $1,454 ($2,282 in 2025 US dollars) for the Incredible Years program, while Frey et al. (2019) found per child costs of $2,716 (with incremental costs per child served by First Steps of $1,911). Given the total per teacher-student dyad ($2,866 for the first student in a classroom) and incremental student costs ($101 for each additional student up to two students in an already treated classroom) found in the current study, if there were two students receiving BEST in CLASS-Elementary in a classroom the per student cost would be $1,484; if three students in a classroom (the maximum served by the program in the current study) the cost per student would be $1,023.

In general, the cost estimates for implementation of BEST in CLASS-Elementary in the current study are generous and should be considered as such. For example, we included costs associated with a program manager (e.g., 16.8% of total cost), teacher recruitment for our research study (e.g., 16.2% of total cost) as well as costs associated with travel to schools for research staff (e.g., 11.4% of total cost), to name but a few. The true implementation costs to a school division may be substantially lower, particularly if they are using school-based coaches to support teacher implementation and have an administrator that can support adoption and installation. Importantly, the training cost per teacher would decrease as additional teachers are added as training is a fixed, one-time cost. That said, this cost savings is not offset by additional teachers being trained due to the added cost of coaching supports, which is the highest expense.

Related to the cost of coaching, approximately one quarter of these costs are associated with pre-intervention training. Consequently, training fewer coaches would also result in cost savings, but at the same time coaches would then likely need to coach more teachers, creating resource challenges in schools. In addition, transportation costs are over 10% of the total cost; as such, identifying site-based coaches may result in additional savings. However, many schools do not have the workforce capacity to take on additional responsibilities such as coaching, so another strategy to reduce transportation costs might be virtual options.

The popularity and common use of web-based platforms (e.g., Zoom; Microsoft Teams) create more opportunities for remote coaching. In a study of web-based training and coaching of the BEST in CLASS-Pre-K program, Conroy et al., (2022a, b) found that the web-based version was as efficacious as the on-site version in reducing child problem behavior. In another study on the web-based version of BEST in CLASS-Pre-K Conroy et al., (2022a, b) found similar levels of teacher fidelity of implementation of BEST in CLASS practices, although teachers in the in-person version had higher self-efficacy than teachers in the web-based condition. Importantly, Conroy et al., (2022a, b) noted that the time (coach training, coaching meetings) and travel costs were less in the web-based version compared to the in-person version, although materials costs (primarily technology devices and resources) were significantly more for the web-based version. While these technology costs are one-time costs, more research is necessary to determine any potential cost savings for remote delivery of interventions such as BEST in CLASS that provide teacher training and implementation supports targeting reductions in SEB problems.

### Limitations

While the findings from this study are informative, there are several limitations that should be considered while interpreting these data. First, this study examined costs from a research study associated with implementation of one program in two states, and as such findings can only generalize to BEST in CLASS-Elementary and these two states. That said, the sensitivity analysis does provide some idea of program costs in other states in the USA. Further, we did not model alternative implementation scenarios such as varying coach-to-teacher ratios, different travel expectations, or alternative staffing configurations (e.g., site-based or virtual coaching). These factors represent implementation decisions rather than fixed program requirements and may influence total costs when districts adapt the model for local use. The wage-based sensitivity analysis and site-level cost variation presented here provide a practical range of expected costs; however, local adaptations to staffing structure, coach caseloads, or travel logistics may increase or decrease costs depending on district capacity and resources. Second, we assumed that school space was available at no cost and required few additional equipment purchases (e.g., technology), consistent with how the program operated in our sites. Districts with space constraints, rental needs, or requirements to purchase new equipment may face higher total costs. Because we did not model these alternative facility scenarios, our estimates may understate costs in contexts where space or equipment expenses are substantial. Third, coach hours were drawn from time sheets, whereas program manager activities were reconstructed from retrospective logs and calendars. Both sources are subject to misreporting and recall error, which could bias input quantities upward or downward. Although we applied standardized rules (e.g., allocating one hour per week to supervision) to enhance consistency, measurement error remains a limitation of the cost estimates. Additionally, our estimates relied on mileage reimbursements at prevailing state rates, but transportation expenses are sensitive to local conditions and the price of gasoline. Because fuel prices are volatile, costs could fluctuate substantially across settings and over time. As a result, our estimates may either overstate or understate transportation expenses depending on the timing and context of implementation. Finally, the costs associated with the implementation of the research project are included in this cost analysis and some of these costs may not be applicable to schools that decide to adopt and sustain BEST in CLASS-Elementary as an intervention program for students with SEB needs. For example, from an implementation science perspective, there are often start-up costs, such as training, when adopting an intervention. However, once the adoption and installation costs have occurred, costs associated with implementation and sustainment can be reduced as capacity is built within the school and classroom.

## Implications and Conclusions

This study provided cost estimates for implementing BEST in CLASS-Elementary, a Tier 2 prevention program. Cost analysis studies across prevention programming in schools are needed to help assist schools in selecting programs and remove cost uncertainty as a barrier to adoption, particularly in a climate where students’ SEB needs are high and school resources to address these needs are diminishing. More research is also needed on approaches that provide cost savings, such as remote coaching, that may help scale effective prevention programming to best support teachers and their students’ SEB needs. Finally, cost-effectiveness studies are sorely needed to help connect implementation costs with child and student outcomes. Better understanding the relationship between costs and outcomes may provide important information for policy makers as they allocate funding for school-based prevention programming.

## Data Availability

NA.
